# Factors Associated with Adherence to Diabetic Retinopathy Screening Among Patients Attending a Nurse-Led Community Clinic in Australia: A Qualitative Study

**DOI:** 10.3390/nursrep15010023

**Published:** 2025-01-14

**Authors:** Fouziah Almouqati, Judith Daire, Catherine Catanach, Jean-Louis deSousa, Sam Quill, Mohamed Estai

**Affiliations:** 1 School of Population Health, Faculty of Health Sciences, Curtin University, Bentley 6102, Australia; f.almouqati@postgrad.curtin.edu.au (F.A.); judith.daire@curtin.edu.au (J.D.); 2 North Metropolitan TAFE, Mt Lawley 6050, Australia; catherine.catanach@nmtafe.wa.edu.au; 3 Department of Ophthalmology, Royal Perth Bentley Group, Perth 6000, Australia; jean-louis.desousa@health.wa.gov.au; 4 Institute of Cardiovascular Science, University College London, London WC1E 6DD, UK; 5 The Australian e-Health Research Centre, CSIRO, Floreat 6014, Australia; 6 School of Human Sciences, The University of Western Australia, Perth 6009, Australia

**Keywords:** diabetic retinopathy, mass screening, patient compliance, health knowledge, attitudes, practice, primary prevention, nurses, community health, health services accessibility

## Abstract

**Background/Objectives:** Despite the availability of screening services, the rate of diabetic retinopathy (DR) screening continues to be suboptimal in Australia, necessitating improvement. However, improving DR screening rates requires a more comprehensive understanding of the factors influencing adherence to the screening recommendations. This study aimed to explore the factors that influence adherence to DR screening among people with diabetes attending a community screening clinic in Australia. **Methods:** This qualitative study included purposively patients with diabetes recruited from a nurse-led community screening clinic in Australia. Semi-structured interviews were conducted to explore barriers and enablers impacting patient adherence to DR screening recommendations. The interview data were analyzed thematically using NVivo based on the socio-ecological model, with salience identified by the frequency of the theme. **Results:** A total of 22 participants completed the interview, including 10 females with a mean age of 60 ± 16.2 years. The interviews identified several factors that improved adherence to DR screening guidelines, including (a) knowledge of the connection between DR and diabetes and the importance of the screening, (b) the care provider’s recommendations, and (c) pre-booked appointments and automatic invitations. Beyond these factors, clinic staff interactions, family support, fear of vision loss, flexible clinic hours, and transportation accessibility also facilitate DR screening adherence. **Conclusions:** The present study identified key multi-level factors influencing adherence to DR screening. While these findings from a single clinic provide valuable insights to inform screening strategies, larger multi-center studies are needed to validate their broader applicability across diverse healthcare settings and populations.

## 1. Introduction

Diabetes has become a major global public health challenge, with projections that its prevalence will approach epidemic proportions in coming decades [1]. According to the International Diabetes Federation, about 700 million people worldwide are expected to have diabetes by 2045 [2]. Uncontrolled diabetes can lead to debilitating microvascular complications such as diabetic retinopathy (DR), nephropathy, neuropathy, and macrovascular complications such as cardiovascular, cerebrovascular, and peripheral vascular diseases [3]. Of the approximately 1.2 million individuals currently living with diabetes in Australia, up to 30% are affected by DR [4].

DR is a progressive retinal microvascular disorder resulting from chronic hyperglycemia-induced damage to the small blood vessels supplying the retina [5]. It is a leading cause of vision impairment and blindness and is responsible for approximately 2.3% of global blindness cases [6]. Early stages of DR are typically asymptomatic, with visual symptoms appearing as the disease progresses, indicating that early detection through regular screening is critical [7]. Timely treatment can prevent up to 90% of vision loss caused by DR [8]. However, if left undiagnosed and untreated, DR can progress to proliferative disease or macular edema, causing severe irreversible vision damage [5].

Regular screening can detect retinal changes early in the disease’s progression, thus, facilitating timely and effective treatment [9]. In Australia, clinical guidelines recommend DR screening at diabetes diagnosis, then every 1–2 years based on risk factors [10]. To improve people’s adherence to DR screening recommendations, the Australian government has undertaken various initiatives. For instance, the government introduced two Medicare Benefits Schedule (MBS) items to support DR screening by general practitioners (GPs). However, screening participation remains suboptimal, with rates of 77.7% in non-Indigenous Australians and 52.9% in Indigenous Australians based on a 2016 national survey [11]. This highlights the need to understand the factors that influence adherence to DR screening among patients with diabetes.

Few studies have explored this topic within Australian populations. Two studies examined patients’ perspectives, identifying individual and interpersonal enablers/barriers related to knowledge, optimism, social influence, age, and beliefs [12,13]. Two others have highlighted the environmental and healthcare system factors influencing screening access and uptake [14,15]. However, no study has provided a comprehensive description of organizational and environmental factors, together with individual, socioeconomic status, or interpersonal factors, to investigate which factors are likely to influence adherence.

The present study used the socio-ecological model (SEM) as its theoretical framework, recognizing that health behaviors, like screening adherence, are influenced by many interacting factors at the individual, interpersonal, organizational, and environmental levels [16]. The SEM provides a comprehensive way to understand these multi-level influences on DR screening behavior. Through a qualitative approach, the current study aimed to explore the various factors that affect adherence to DR screening among individuals with diabetes who attended a dedicated DR screening clinic in Perth, Australia. While this exploratory design focuses on gaining rich, contextualized insights rather than statistical generalization, it offers valuable information to help improve screening strategies.

## 2. Materials and Methods

### 2.1. Study Setting

The present study was conducted at the Roaming Education and Community Health (REACH) clinic in Perth, Western Australia, over the period of January–March 2022. REACH is a community-based clinic led by supervised TAFE nurse students initiated in 2012, providing health education and undertaking preventative health checks for patients, including DR screening [17]. The Diabetic Retinopathy Screening service at the REACH is a hospital outpatient clinic, operated by the North Metropolitan TAFE staff and collaborates closely with Royal Perth Hospital’s Ophthalmology department.

### 2.2. Study Design

This was a qualitative study that used a semi-structured interview of patients attending REACH clinic. A descriptive qualitative content analysis approach, guided by the SEM framework and naturalistic inquiry, was used to interpret meaning from data generated from the participants’ responses [18]. This method was appropriate due to its advantage in systematically and objectively analyzing a varied amount of qualitative data to understand the phenomena of interest [19]. The reporting of this study followed the Standards for Reporting Qualitative Research (SRQR) (Table S1) [20].

### 2.3. Researcher Characteristics and Reflexivity

As a young research scholar without living experience of diabetes, the primary researcher (FA) position may have influenced participant interactions and data interpretation. To mitigate potential biases, the researcher engaged in reflexive practices such as journaling and regularly discussing with the diverse team to challenge assumptions and consider alternative perspectives.

### 2.4. Theoretical Framework

**Figure 1:**
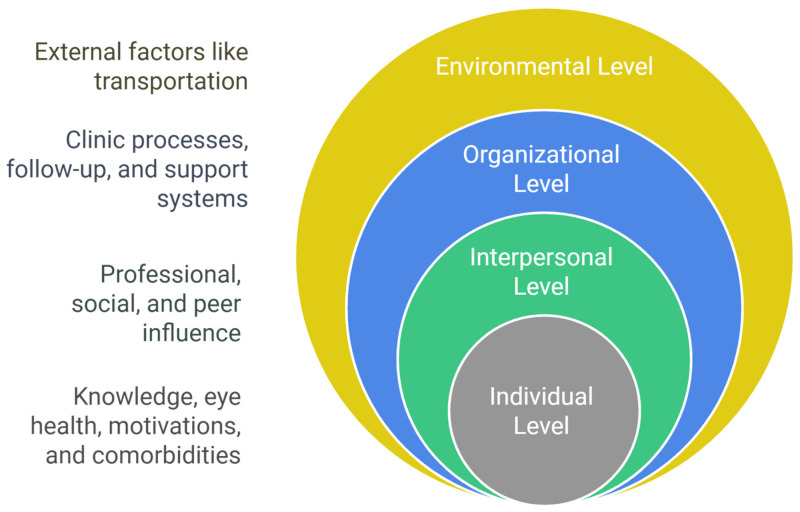
Socio-ecological model illustrating interactions of patients with diabetes with their environment at each level.

According to the SEM, health behavior and utilization of DR screening in this context is an outcome determined by the interaction of factors at various levels [16]. The individual in this model was placed in the center and surrounded by various systems [21]. This theory states that the microsystem (individual level) has the strongest influence. These factors are directly related to the individual, such as age, other diseases, knowledge, beliefs, etc. The second system is the mesosystem (interpersonal level), which looks beyond the individual characteristics of a person’s relationship and interaction with others, such as family, friends, colleagues, and healthcare professionals. The ecosystem (organizational level) exerts indirect positive/negative influences, including the impact of formal and informal rules and regulations. Finally, the macrosystem (environmental level) includes the impact of the built environment and availability of transportation (Figure 1).

### 2.5. Participants

Purposive convenience sampling was used to recruit the participants. We aimed to obtain a sample representing diverse demographics and adherence levels. The eligibility criteria were as follows: patients diagnosed with diabetes, aged >18 years, who attended the REACH clinic, able to provide informed consent, and English or Arabic speaking. Patients visiting the REACH clinic for DR screening are advised to wait until the side effects of the eye drops improve, which takes approximately one hour. The primary investigator approached potential participants during this waiting period, introduced the study using a participant information consent sheet and invited them if they would like to participate.

Participant recruitment continued until data saturation was reached, with no new themes emerging from additional interviews [22].

### 2.6. Data Collection

Data on the participants’ demographic characteristics and adherence status were collected from the clinic records. Adherence status was categorized as adherent or non-adherent based on their eye screening history and recommended schedules.

Qualitative data were collected through semi-structured interviews to explore the factors influencing adherence across individual, interpersonal, organizational, and environmental levels. The interview topic guide (Table S2) was developed by the first author based on a literature review and informed by the SEM. The co-authors (JD and ME) reviewed and edited the topic guide until a consensus was reached. The interview topic guide was then presented to a consumer member, who reviewed the language and structure of the questions, after which edits were made based on their feedback. Face-to-face individual interviews were conducted by the primary investigator at the REACH clinic after the participants had finished their eye screenings. The interviews lasted up to 39 min. To ensure accuracy, all interviews were recorded using an audio recorder and professionally transcribed thereafter.

### 2.7. Data Analysis

A deductive content analysis approach guided by the SEM framework was used to analyze the interview data as a basis for coding and theme development [18,23]. The coding framework for this study was developed deductively by authors (JD and FA) based on SEM. The first author (FA) extensively familiarized themselves with the interview transcripts and created a categorization matrix containing notes, summaries, and reflections on each interview to ensure a clear overview of the data for the research team. Interview transcripts were double-checked for accuracy and imported into the NVivo version 13 (QSR International Pty Ltd., Burlington, MA, USA, 2020). To determine the importance of subthemes, repeated references to one theme by one respondent were counted as one utterance to avoid inflation of the theme’s importance. Finally, the coding and importance of themes were reviewed by JD, and any disagreement was resolved through consensus discussions.

### 2.8. Rigor

Strategies to ensure rigor included method and data source triangulation, expert and consumer validation of the interview topic guide, verbatim transcriptions, and journaling reflections between interviews. To minimize potential biases, several strategies were employed, including building rapport with participants, using probing questions to verify responses, and conducting coding and analysis independently by two researchers, with discrepancies resolved through discussion.

## 3. Results

### 3.1. Sociodemographic Characteristics and Adherence Status

A total of 22 participants were enrolled in this study, of whom 19 (86%) were adherent and 3 (14%) were non-adherent to DR screening. There were 12 men (54.54%) and 10 women (45.45%). Participants’ ages ranged from 23 to 80 years, with a mean of 60 ± 16.2 years. The mean duration of diabetes was 16.2 ± 11.9 years. The ethnicity was classified as Oceanian (50%), Asian (36.36%), North African/Middle Eastern (9.09%), and European (4.54%). In terms of educational attainment, 18.18% of the participants had attained primary education, 9.09% secondary education, 36.36% high school education, and 36.36% tertiary education. Most participants (81.81%) had Type 2 diabetes and 18.18% had Type 1. Lastly, 77.27% of the participants had comorbidities other than diabetes, and only 40.9% had eye problems or had experienced them (Table 1).

### 3.2. Enablers and Barriers to DR Screening Adherence

#### 3.2.1. Individual-Level Factors

Knowledge: Knowledge about the connection between diabetes and DR plays a crucial role in DR screening adherence. Most participants (*n* = 15) showed basic knowledge that diabetes can lead to vision loss, providing examples like “lose your sight” (P10_adherent). Some showed good understanding of how diabetes affects the eyes, quoting “the main problem is the blood supply to the back of eyes” (P20_adherent). However, few participants were unaware of the impact of diabetes on the eyes. One quoted “I did not think it would be as serious a thing to my eyes, I just put it down to old age” (P06_non-adherent).

**Figure 2:**
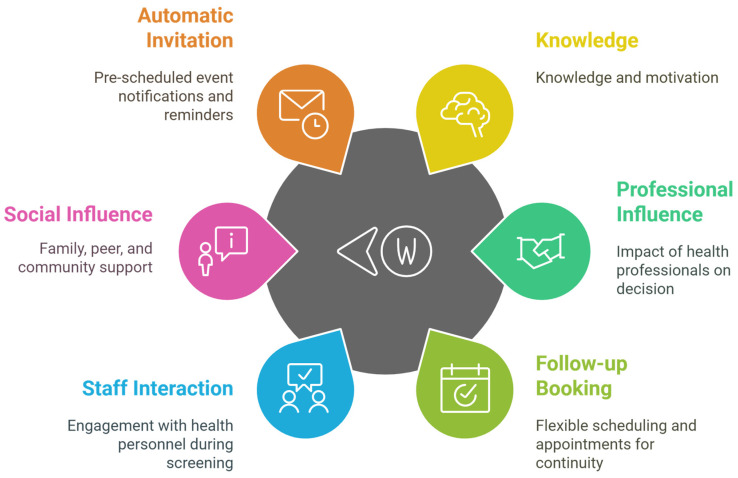
Illustration of main factors influencing DR screening adherence.

Regarding awareness of screening importance, most knew regular eye checks should be routine. Some recognized that screening allows early detection, quoting it “gives a heads up” or provides a “warning you’re doing something wrong” (P20_adherent). In contrast, a few participants were unaware of the importance of regular DR screening. Their responses focused on general eye protection like “Use eye protection and stay out of the sun” (P19_non-adherent) rather than on screening specifically (Figure 2).

Eye health: Having existing eye issues or undergoing eye treatment encouraged some participants to adhere to DR screening. One quoted “I have to go because I cannot see things very properly” (P12_adherent). Another said, “After my cataract operation for both eyes, I have been undergoing these one-and-a-half year checks” (P09_adherent). In contrast, not experiencing any vision symptoms was a barrier for one participant who felt no need for regular screening since their vision seemed fine, saying “my eyes have been really good in general...they still feel pretty good” (P04_non-adherent).

Comorbidities: Among the 17 participants with health issues besides diabetes, only 1 expressed a link between other diseases and DR screening adherence. Having other diseases helped participants adhere to screening by making them more familiar with health checks in general and more aware of their importance. They explained “I am used to health checks...for cancer, for the heart, so it is just another one. So now I just know that if you do not get it checked, it can be too late” (P20_adherent).

Motivations: The fear of going blind and family love were enablers for three participants. One participant (P07_adherent) expressed, “I would hate to lose my eyesight for so many different reasons: the loss of independence”. Another (P15_adherent) emphasized staying healthy for their family’s sake, stating, “I have a family. I have kids. I want to see them grow up, get married, and maybe have grandchildren. Therefore, I want to see that that future becomes a reality. I think the only thing I can do is to be healthy”.

#### 3.2.2. Interpersonal-Level Factors

Professional influence: Health professionals played a key role in enabling access to and adherence to DR screening for many participants. The majority cited being referred by their GPs or endocrinologists as the reason they underwent screening, with one quoting “My GP said it; he referred me to eye specialist, and that is why I have done it every year” (P11_adherent). Others noted additional referral sources, such as nephrology specialists, cancer doctors, and diabetes educators. In contrast, one non-adherent participant cited a lack of professional recommendation as a barrier, explaining that “it just seemed like [screening] did not come up, or maybe there was some miscommunication” with their specialist (P19_non-adherent).

Staff interactions: Positive interactions with REACH staff shaped adherence for some participants by making them feel comfortable and at ease in the screening environment. This was commonly expressed, with one quoting “I always come here without apprehension, and I enjoy coming here” (P21_adherent). The staff caring approach appeared to facilitate continued compliance with DR screening.

Professional assistance: One adherent participant with mobility limitations and a language barrier had access to DR screening, enabled by support from a professional career. When asked why they attended Arabic, they explained “My career. I showed her the letter...and she brought me here today” (P02_adherent). This exemplifies the vital assistance that a healthcare worker can provide to facilitate screening for high-need patients.

Social influence: Family and friends played a multifaceted and influential role in enabling adherence for some participants. For those with language barriers or forgetfulness, total dependence on family support was critical for facilitating screening attendance. As one explained, “I cannot go by myself, I cannot communicate well. My kids look after me” (P22_adherent). Beyond this, some cited partner motivations a driving factor, with one stating “My wife makes sure I do not miss the appointments” (P17_adherent). Spouses seemingly help to hold participants accountable. Furthermore, some mentioned that family assisted with practical barriers such as transport to appointments, for example, “My wife brought me here and she is waiting outside” (P16_adherent).

Others’ experiences: Seeing vision complications in close ones with diabetes motivated adherence in some participants who expressed not wanting to end up in the same situation. One quoted “My mother ended up legally blind because of diabetes. That is good inspiration for me to try and avoid that” (P07_adherent), exemplifying how witnessing related suffering can enable adherence (Figure 2).

#### 3.2.3. Organizational-Level Factors

Automatic invitation: Many participants accessed the initial DR screening not through the typical route of GP or specialist referrals, but rather through receiving formal invitation letters from the RPH system. Some were unsure how they were identified for DR screening invites, with one quoting “I did not know about it. I had to do the screening once they started sending me the letters” (P17_adherent). Another hypothesized “The diabetic association…sent my information to the hospital” (P21_adherent). This automatic screening invitation system appears to have facilitated access for many people (Figure 2).

Follow-up booking: Most participants expressed acceptance of or support for the system where REACH books follow-up appointments based on screening results, rather than relying on patient self-scheduling. Some noted that this approach facilitated adherence, with one explaining “Otherwise, I will forget because it is a year apart” (P20_adherent). Removing the need for patients to remember the self-book seems to promote continued screening compliance.

Clinic flexibility: Among those who had no objection, three stated that their initial appointment had not suited them, so they had to rebook but were happy that they had not waited too long. One said: “I was really lucky with my first referral when I rebooked. It was like a month away, and then they rebooked it for me, and that was maybe for two weeks” (P04_non-adherent).

Waiting time: A short waiting time before seeing a specialist was an enabler for one participant to adhere to DR screening. They said: “If I have an appointment at 8:30, at 8:30, I am being served” (P15_adherent).

Reminder message: Appointment letters for the REACH clinic are sent to patients approximately three to six weeks before the appointment date. Four participants indicated that they depended on the messages to remember their appointment, for example, “I wait until I get a message about this” (P10_adherent).

Medicare coverage: One participant indicated that the fact that they did not need to pay out of pocket was an enabler for them to access the screening and “not putting it off” (P04_non-adherent).

#### 3.2.4. Environmental-Level Factors

Access to public transportation was cited as an enabler of adherence to DR screening for the three participants. One stated: “I usually board the bus. It is very convenient: only one ride” (P15_adherent).

### 3.3. Factors Most Likely to Influence Adherence to DR Screening

**Figure 3:**
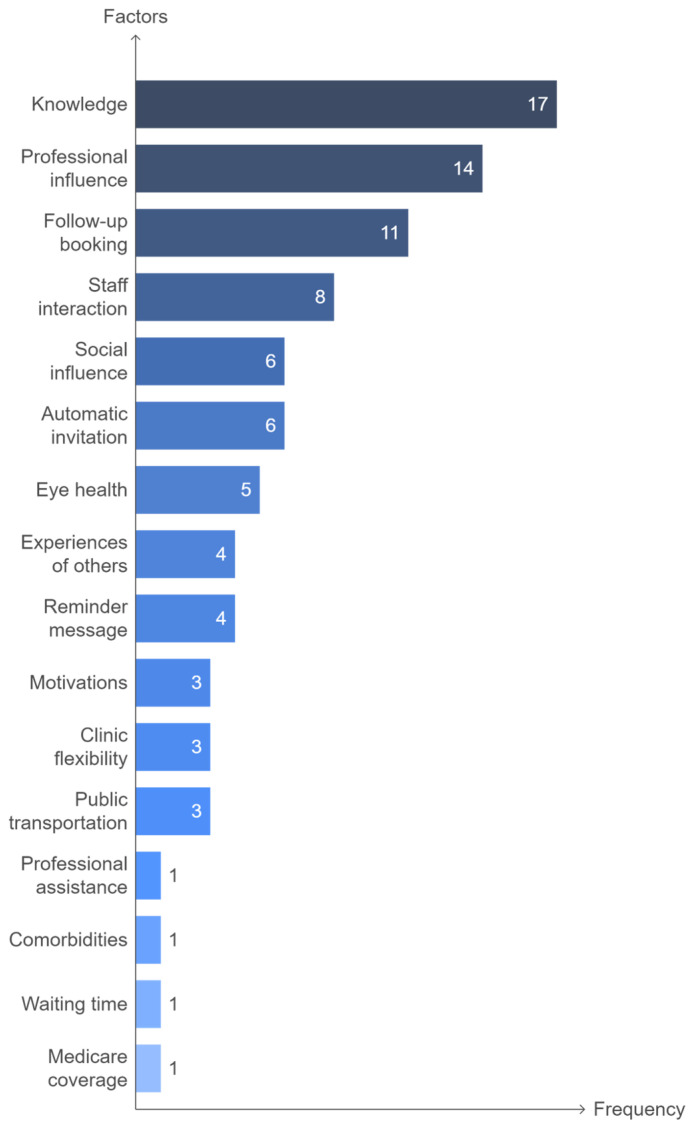
Ranking of factors influencing DR screening adherence.

The most influential factors were knowledge (17 participants), professional influence (14), follow-up booking method (11), staff interaction (8), social influence and automatic invitation (6 each), eye health (5), experiences of others and reminder messages (4 each), motivations, clinic flexibility and access to public transportation (3 each), comorbidities, professional assistance, short waiting time, and Medicare coverage (1 each) (Figure 3).

The relative importance of factors was determined by the total frequency of mentions across all interviews, where repeated references to one theme by a single respondent were counted as one instance to avoid inflation. Factors were then ranked from 1 (most frequently mentioned) to 9 (least frequently mentioned) based on their total frequency count combining both enabler and barrier mentions.

## 4. Discussion

The current study explored the factors that influence adherence to DR screening and yielded important results. Participants frequently cited individual-level knowledge of diabetes complications and the importance of screening as the most influential factors for adherence. Other key enablers include professional advice to seek DR screening, appointment-booking methods, automatic screening invitations, and family/friend support.

The primary barriers were lack of knowledge and professional screening advice. Knowledge regarding DR risks, complications, and screening benefits being the most influential enabler, which aligns with a previous Australian study highlighting patient awareness of asymptomatic progression of DR, and the value of screening enables adherence prioritization [12]. Our finding that lack of knowledge was a key barrier concurs with evidence of misconceptions about the impact of diabetes on eye hinder screening uptake [13]. The importance of knowledge comes from the fact that most of the adherent participants were aware of the connection between diabetes and DR, and most of those who were non-adherent showed misconceptions. A study conducted in the UK also showed that understanding the consequences of DR enables patients to prioritize protecting their eyes and, therefore, contributes to better uptake [24]. Experiencing eye problems was an enabler that contributed to some of our participants’ adherence, which was also observed in a previous study [25].

Professional DR screening recommendations were a major facilitator that corroborates extensive research showing that GP’s advice is pivotal in catalyzing optimal DR screening adherence [26,27]. This reinforces the guidelines urging Australian healthcare providers to discuss DR with patients with diabetes [10].

Besides knowledge and professional recommendations, social support and professional assistance play a central role in facilitating adherence to DR screening. For example, practical support from family or friends with transportation, help with reminders of appointments, or social pressure to participate in screening would positively affect participation in DR screening. These results are consistent with previous reports that highlighted the importance of family involvement and peer support in self-management behaviors [28,29,30]. Another such noted enabler is professional assistance from healthcare workers, particularly for those with mobility limitations or language barriers [30,31]. This, therefore, underscores the need for tailored support for more vulnerable populations concerning increasing DR screening. Other enablers described include positive clinic staff behaviors, flexible hours, and available transport [30]. These findings indicate, therefore, that any effective intervention proposed for the improvement of DR screening adherence will need to adopt a comprehensive, multilevel approach in addressing patient, care provider, and environmental factors.

The present study highlights several organization-level enablers. Appointment bookings and invitations easing barriers for patients reflect previous findings on facilitators, such as sending pre-booked appointments and patient reminders [24]. Our study emphasizes the importance of initiating screening without relying on patient-initiated appointments. In addition, the findings of this study indicate that accessibility and convenience are significant factors that promote adherence to DR screening. Although our research primarily focused on a community-based clinical model, emerging evidence suggests that telemedicine and home-based screening may further enhance participation in screening [32,33]. Future research should evaluate the feasibility and effectiveness of implementing these alternative screening approaches to complement the existing services and improve the coverage of DR screening.

This study has several important implications and provides recommendations for practice and policy as the followings:

- Lack of knowledge regarding diabetes complications and the importance of screening are major barriers. This highlights the need for more patient education regarding DR risks and screening benefits, especially focusing on high-risk groups. Educational resources and counseling should be provided at diagnosis and integrated into the ongoing diabetes care.
- Healthcare provider recommendations were the second most influential factor enabling screening adherence. This underscores the pivotal role of healthcare providers in catalyzing optimal adherence. Policies are required to prompt clinicians to encourage patients to undergo DR screening during every diabetes encounter. Education and reminder systems could facilitate this process.
- Organizational factors, such as pre-booked screening appointments and reminders were significant facilitators. Policies expanding the implementation of these system supports, rather than relying solely on patient-initiated care, could optimize follow-up screening and convenience.
- Dedicated screening programs with coordinated appointment systems help convey the importance of routine DR surveillance to patients. Scaling up programs across underserved areas can improve adherence.

The present study has several important limitations. Recruiting participants from a single clinic through purposive convenience sampling may introduce selection bias, which limits the generalizability of our findings to other screening settings. Self-reported data could be subject to recall bias regarding factors influencing adherence, as well as social desirability bias, potentially leading to underreporting of non-adherence. Although our sample size allowed us to reach thematic saturation, the small number of non-adherent participants (*n* = 3) restricted our ability to fully understand the barriers to screening access for this important subgroup.

While our findings provide valuable insights, their feasibility and scalability require careful evaluation before widespread implementation. Future research should validate these findings through larger multicenter studies with more diverse populations, particularly nonadherent patients and those from rural areas. Mixed-method approaches would help assess practical implementation considerations and strengthen the evidence base for improving adherence to DR screening. Additionally, further qualitative studies are needed to explore healthcare providers’ perspectives on enhancing adherence to DR screening.

## 5. Conclusions

The present study offers insights into the factors influencing adherence to DR screening among patients attending a community-based clinic. It highlights several important factors that may affect screening behavior, including patient knowledge about diabetes-related eye complications, healthcare provider recommendations, and organizational support, such as pre-booked appointments and reminder systems. Although these findings suggest potential areas for intervention, further research through larger multicenter studies across diverse populations is needed to validate and extend these recommendations. Such evidence would provide a stronger foundation for developing and implementing strategies to optimize DR screening programs and prevent avoidable vision loss in people with diabetes.

## Figures and Tables

**Table 1:** Demographic characteristics of participants.

Variables	Total (*n* = 22)
Sex	
Male	12 (54.54%)
Female	10 (45.45)
Mean age in years (±SD)	60 ± 16.2
Mean duration of diabetes in years (±SD)	16.2 ± 11.9
Type of diabetes	
Type 1 diabetes	4 (18.18%)
Type 2 diabetes	18 (81.81%)
Ethnicity	
Oceanian	11 (50%)
Asian	8 (36.36%)
European	1 (4.54%)
North African/Middle Eastern	2 (9.09%)
Education	
Primary school	4 (18.18%)
Secondary school	2 (9.09%)
High school	8 (36.36%)
Tertiary	8 (36.36%)
Comorbidities	
Yes	17 (77.27%)
No	5 (22.72%)
Eye problems	
Yes	9 (40.9%)
No	13 (59.09%)

## Data Availability

The data for this study cannot be shared, as we do not have permission from the participants or ethics approval to do so.

## Supplementary Materials

The following supporting information can be downloaded at: https://www.mdpi.com/article/10.3390/nursrep15010023/s1, Table S1: SRQR checklist; Table S2: interview topic guide.

## Public Involvement Statement

Public involvement in this study included consultation with a consumer who had lived experience of diabetes, providing valuable insights into the study’s design and interpretation of findings. The consumer reviewed the interview topic guide and provided feedback on the language and structure of questions before data collection commenced. They also contributed to the analysis by reviewing initial findings to ensure the research reflected the experiences of people with diabetes.

## Guidelines and Standards Statement

This manuscript was drafted against the Standards for Reporting Qualitative Research (SRQR) [20].
