# Supplementary material for: Factors Associated with Adherence to Diabetic Retinopathy Screening Among Patients Attending a Nurse-Led Community Clinic in Australia: A Qualitative Study
Source: Nurs Rep. 2025 Jan 14;15(1):23. doi: 10.3390/nursrep15010023 (PMC11767495; doi:10.3390/nursrep15010023)
Supplement: Supplementary file 1 [file nursrep-15-00023-s001.zip › nursrep-3295165-supplementary.pdf]

Table S1. Standards for Reporting Qualitative Research (SRQR)

O'Brien B.C., Harris, I.B., Beckman, T.J., Reed, D.A., & Cook, D.A. (2014). Standards for reporting qualitative research: a synthesis of recommendations. Academic Medicine, 89(9), 1245-1251.

No	Topic	Item	Page No
**Title & abstract**			
S1	Title	Concise description of the nature and topic of the study Identifying the study as qualitative or indicating the approach (e.g., ethnography, grounded theory) or data collection methods (e.g., interview, focus group) is recommended.	1
S2	Abstract	Summary of key elements of the study using the abstract format of the intended publication; typically includes background, purpose, methods, results, and conclusions.	1
**Introduction**			
S3	Problem formulation	Description and significance of the problem/phenomenon studied; review of relevant theory and empirical work; problem statement.	3-4
S4	Purpose or research question	Purpose of the study and specific objectives or questions.	4
**Methods**			
S5	Qualitative approach and research paradigm	Qualitative approach (e.g., ethnography, grounded theory, case study, phenomenology, narrative research) and guiding theory if appropriate; identifying the research paradigm (e.g., postpositivist, constructivist/ interpretivist) is also recommended; rationale.	4

S6	Researcher characteristics and reflexivity	Researchers' characteristics that may influence the research, including personal attributes, qualifications/experience, relationship with participants, assumptions, and/or presuppositions; potential or actual interaction between researchers' characteristics and the research questions, approach, methods, results, and/or transferability.	4
S7	Context	Setting/site and salient contextual factors; rationale.	4
S8	Sampling strategy	How and why research participants, documents, or events were selected; criteria for deciding when no further sampling was necessary (e.g., sampling saturation); rationale.	5
S9	Ethical issues pertaining to human subjects	Documentation of approval by an appropriate ethics review board and participant consent, or explanation for lack thereof; other confidentiality and data security issues.	4
S10	Data collection methods	Types of data collected; details of data collection procedures including (as appropriate) start and stop dates of data collection and analysis, iterative process, triangulation of sources/methods, and modification of procedures in response to evolving study findings; rationale.	5-6
S11	Data collection instruments and technologies	Description of instruments (e.g., interview guides, questionnaires) and devices (e.g., audio recorders) used for data collection, if/how the instrument(s) changed over the course of the study.	5-6

S12	Units of study	Number and relevant characteristics of participants, documents, or events included in the study; level of participation (could be reported in results).	6
S13	Data processing	Methods for processing data prior to and during analysis, including transcription, data entry, data management and security, verification of data integrity, data coding, and anonymization/deidentification of excerpts.	6
S14	Data analysis	Process by which inferences, themes, etc., were identified and developed, including the researchers involved in data analysis; usually references a specific paradigm or approach; rationale.	6
S15	Techniques to enhance trustworthiness	Techniques to enhance trustworthiness and credibility of data analysis (e.g., member checking, audit trail, triangulation); rationale.	6
**Results**			
S16	Synthesis and interpretation	Main findings (e.g., interpretations, inferences, and themes); might include development of a theory or model, or integration with prior research or theory.	6-10
S17	Links to empirical data	Evidence (e.g., quotes, field notes, text excerpts, photographs) to substantiate analytic findings.	-
**Discussion**			

S18	Integration with prior work, implications, transferability, and contribution(s) to the field	Short summary of main findings; explanation of how findings and conclusions connect to, support, elaborate on, or challenge conclusions of earlier scholarship; discussion of scope of application/ generalizability; identification of unique contribution(s) to scholarship in a discipline or field.	10-13
S19	Limitations	Trustworthiness and limitations of findings.	12
S20	Funding	Sources of funding and other support; role of funders in data collection, interpretation, and reporting.	13
S21	Conflicts of interest	Potential sources of influence or perceived influence on study conduct and conclusions; how these were managed.	13

Table S2. Interview Topic Guide

Participant DoB:	
Gender:	
Ethnicity:	
Level of education:	
Interview code:	
Participant pseudonym:	

Interviewer: *thank the participant for their time and restate the research information.*
Warm-up questions:

1. **In your own words, can you please tell me what diabetes is?**

Probe:

- Do you know what type of diabetes you are diagnosed with?
- How long have you been living with diabetes?

2. **What do you know about the long-term complications of diabetes?**

Prompt if the participant did not specify visual impairment (diabetic retinopathy):

- Have you heard about the term diabetic retinopathy before?
- If Y: can you please tell me what do you know about it?

3. **How do you think a person who is living with diabetes should take care of their eyes?**

Prompt:

- What about regular fundus screening?

Probe if necessary:

- What do you think the benefit of regular eye screening is for a person living with diabetes?

4. **Have you done this screening before this time?**

Probe:

- How many times?
- Do you remember when the last one was? (Prompt for estimation)
- Where did you do your eye screening(s)? (Prompt for full list)
- If it was done in another clinic: Do you remember how the procedure was?

Sometimes our plans are hindered by things and circumstances that are out of our control.

5. Has it happened to you that you could not do your eye screening at the time you planned?

Probe:

- Do you remember when?

The rest of the questions will focus on factors that we as researchers think may have helped and hindered you from getting regular eye screening. However, our knowledge in this aspect is not complete. Thus, we seek to know the views of a person who is actually living the experience. Therefore, I was hoping you could help me learn which of the following factors are related to you and how they have affected you to get regular eye screening.

6. Can you please tell me how did you know that you need to do regular eye screening?

Probe if necessary:

- Have you had a discussion with a healthcare professional (GP/ endocrinologist) about it?
- What were your thoughts on your GP recommendations at that time?
- Did you attend diabetes education visits?
- Did you get reading materials (brochures or leaflets)?
- If Y: how did you access them?

7. Think about your past appointments, do you think the interaction with the screener (and clinic staff) affected your decision to attend an upcoming screening appointment?

Probe:

- What did you like/dislike about their interaction?

8. Do you think the family, friends, work colleagues or acquaintances have had a role in your past journey with regular eye check ups?

Probe:

- If (1), What kind of support have been provided?
- If (2), tell me how they affected you?
- Was there any kind of support you wish to get from the people you interact with in your social/work life?

9. Think about your other commitments, do you think they impact(ed) you from getting regular eye screening?

Probe:

- Can you tell me what these commitments are/were?

10. Can you please tell me how your previous and current appointment has been booked?

Probe:

- Were they booked by you or GP/clinic/someone else?

11. Do you think this/these method(s) of booking is related to your regular attendance?

Probe:

- Which one do you prefer? And why?

12. What other aspects related to appointments affected your regular eye screening?

Prompt:

- What about the wait time before the date of the appointment?
- What about the waiting time before the doing screening?
- parking

13. Have you ever delayed getting your eye screening because you have forgotten to book or attend?

Probe if no:

- Can you please tell me what has helped you always to remember your appointments?

14. Have you had (or currently have) any health issues other than diabetes?

Probe if Y:

- Can you tell me what are/were they?

15. Has/have your health issue(s) affected your decision to attend any of your screening appointments?

Probe if (1) or (2):

- Tell me why, please?

16. **Can you please think back to the time that you knew that diabetes could affect your vision, can you describe your thoughts and feelings at that time?**

17. Did these thoughts/feelings affect you from seeking regular eye screening?

18. Can you please think back to when you had an upcoming eye screening appointment, did you have any feelings about this?

Prompt:

- What did you feel?

Probe:

- What is your feeling about the method of screening?
- How did it affect you?

19. Consider your past screening appointments, did you have to pay out of your pocket?

20. **Do you think this method(s) of payment impacted you?**

21. **Considering the distance from your home to this clinic (and the previous if there were any), do you think it is related to your attendance to your screening appointment.**

22. Is there anything else you want to highlight as an aspect that helped you or hindered you from getting regular eye screening?

23. What aspects do you wish healthcare decision-makers would work on with regard to diabetic eye screening?
